# Laser induced spherical bubble dynamics in partially confined geometry with acoustic feedback from container walls

**DOI:** 10.1016/j.ultsonch.2023.106664

**Published:** 2023-10-29

**Authors:** Lei Fu, Xiao-Xuan Liang, Sijia Wang, Siqi Wang, Ping Wang, Zhenxi Zhang, Jing Wang, Alfred Vogel, Cuiping Yao

**Affiliations:** a Institute of Biomedical Photonics and Sensing, Key Laboratory of Biomedical Information Engineering of Ministry of Education, School of Life Science and Technology, Xi’an Jiaotong University, Xi’ an 710049, China; b Institute of Biomedical Optics, University of Luebeck, 23562 Luebeck, Germany; c Zunyi Medical University, Zhuhai Campus, Zhuhai 519041, China; d State Key Laboratory of Transient Optics and Photonics, Chinese Academy of Sciences, Xi’an 710119, Shaanxi, China

**Keywords:** Laser-induced cavitation, Partial confinement, Acoustic feedback, Elastic wall, Vibrations, Extended Rayleigh-Plesset model

## Abstract

We investigated laser-induced cavitation dynamics in a small container with elastic thin walls and free or partially confined surface both experimentally and by numerical investigations. The cuvette was only 8–25 times larger than the bubble in its center. The liquid surface was either free, or two thirds were confined by a piston-shaped pressure transducer. Different degrees of confinement were realized by filling the liquid up to the transducer surface or to the top of the cuvette. For reference, some experiments were performed in free liquid. We recorded the bubble dynamics simultaneously by high-speed photography, acoustic measurements, and detection of probe beam scattering. Simultaneous single-shot recording of radius-time curves and oscillation times enabled to perform detailed investigations of the bubble dynamics as a function of bubble size, acoustic feedback from the elastic walls, and degree of surface confinement. The bubble dynamics was numerically simulated using a Rayleigh-Plesset model extended by terms describing the acoustically mediated feedback from the bubble’s environment.

Bubble oscillations were approximately spherical as long as no secondary cavitation by tensile stress occurred. Bubble expansion was always similar to the dynamics in free liquid, and the environment influenced mainly the collapse phase and subsequent oscillations. For large bubbles, strong confinement led to a slight reduction of maximum bubble size and to a pronounced reduction of the oscillation time, and both effects increased with bubble size. The joint action of breakdown-induced shock wave and bubble expansion excites cuvette wall vibrations, which produce alternating pressure waves that are focused onto the bubble. This results in a prolongation of the collapse phase and an enlargement of the second oscillation, or in time-delayed re-oscillations. The details of the bubble dynamics depend in a complex manner on the degree of surface confinement and on bubble size. Numerical simulations of the first bubble oscillation agreed well with experimental data. They suggest that the alternating rarefaction/compression waves from breakdown-induced wall vibrations cause a prolongation of the first oscillation. By contrast, liquid mass movement in the cuvette corners result in wall vibrations causing late re-oscillations. The strong and rich interaction between the bubble and its surroundings may be relevant for a variety of applications such as intraluminal laser surgery and laser-induced cavitation in microfluidics.

## Highlights

• Evaluation of dynamics for >5000 events with different bubble size and confinement.
• Bubble expansion causes transient ambient pressure rise depending on confinement.
• Breakdown-induced wall vibrations cause alternating stress waves acting back on bubble.
• Feedback produces shortening or prolongation of oscillations, and re-oscillations.
• Good agreement with simulations using Rayleigh-Plesset model plus pressure terms.

## Graphical abstract

**Figure:**
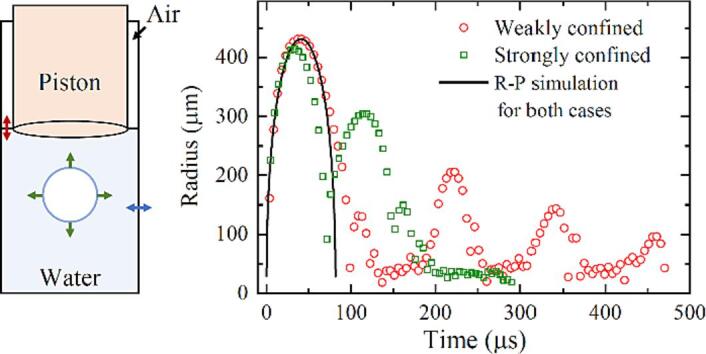

## 1 Introduction

Cavitation bubble dynamics is strongly influenced by the interaction between the bubble and its environment, and this interaction is usually mediated by changes of the pressure–time-history and of the flow-velocity distribution. A ‘pure’ constellation is the oscillation of a laser-induced spherical bubble in an infinite bulk of liquid. Here, the plasma-driven bubble expansion is counteracted by liquid inertia and influenced by liquid compressibility but there is no feedback from any boundary [1], [2], [3], [4]. The opposite extreme is the oscillation of a single bubble driven by an external sound field [5]. Here, energy from the sinusoidal sound waves is converted into a strongly nonlinear spherical bubble oscillation accompanied by spatiotemporal energy concentration and shock wave emission at collapse but the bubble is a passive part and does not change the driving sound field. Between these extremes, there is a range of intermediate constellations that are defined by different kinds of boundaries (or other bubbles) in the vicinity of the bubble that introduce a feedback between the bubble and its surroundings. In the following, we will focus on single laser-induced cavitation bubbles because of their importance for material processing in general and laser surgery in particular [6], [7], [8], [9], [10].

Solid, free, or elastic boundaries on one side of a laser-induced bubble with semi-infinite space on the opposite side act back on the bubble dynamics by influencing the surrounding pressure field and flow velocity distribution [11], [12], [13], [14], [15], [16]. The asymmetry of the boundary conditions induces an aspherical dynamics with jetting, which depends on the type of boundary and the stand-off distance between bubble and boundary [12], [13], [17], [18], [19], [20]. A related case is the unidirectional impact of a shock wave on a bubble [21]. The influence of combinations of boundaries of same or different types that are located on different sides of the bubble has also been investigated [22], [23], [24], [25], [26], [27].

**Fig. 1:**
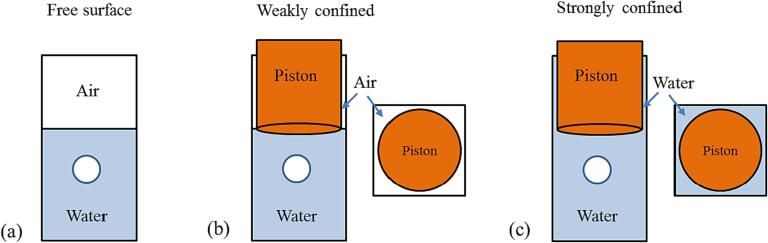
Bubble in a cuvette with elastic walls and different types of confinement at the liquid surface. (a) free surface, (b) weak confinement by a piston with slightly smaller diameter than the container width. The piston end just touches the water surface, and an air gap remains between the cuvette walls and the piston, (c) strong confinement; the gap between cuvette wall and transducer is filled with water.

Containers or tubes with solid or elastic walls introduce a stronger degree of confinement to the bubble dynamics [28], [29], [30]. In the present paper, we investigate the dynamics of laser-induced bubbles in a small (10 mm) water cuvette with elastic walls, where the liquid surface is either free or partially confined by a cylindrical piston immersed into the liquid (Fig. 1). Bubbles are produced on the central axis of the container with square cross section such that their oscillations remain approximately spherical in spite of feedback from the surroundings. The bubble radius is varied between 0.2 and 0.8 mm. Thus, the container is small enough for breakdown events to induce wall vibrations, and bubbles are large enough relative to the cuvette dimensions to experience a significant feedback from these vibrations, which change with different degrees of liquid surface confinement.

Complete sealing of a liquid-filled container with rigid walls or thick elastic walls with large bulk modulus would result in a reduction of maximum bubble size and bubble oscillation time because the bubble expansion increases the ambient pressure surrounding the bubble [31], [32], [33]. In our investigations, the elastic container walls are thin and flexible, and their oscillations produce a more complex feedback on bubble oscillations than a pure bubble-induced pressure rise [34]. The outgoing movement of the container walls that is induced by the laser-induced shock wave and bubble expansion may continue for a while during the bubble collapse, which will produce underpressure or tensile stress prolonging the collapse. The backswing of the container wall also influences the bubble dynamics, depending on the relative phase between wall and bubble oscillations. The feedback strength is enhanced by the central location of the bubble because the pressure waves emanating from the walls interfere at the container center, such that relatively large negative and positive pressure amplitudes are produced. The feedback stress transients are bipolar, with a tensile stress component arriving first.

The top side of the container is either left completely open (‘free surface’), or semi-sealed by a cylindrical piston. The piston is either just dipped into the liquid [Fig. 1(b)], which leads to ‘weak confinement’ of the bubble surroundings, or it is deeply immersed, resulting in ‘strong confinement’ [Fig. 1(c)]. The piston is located in the center of the opening, with spaces at the corners of the container. The laser-induced bubble oscillations induce an up-and-down movement of the liquid in the corners that, depending on the immersion depth of the piston, involves different liquid mass, wall friction, and flow impairment by the piston corner. This goes along with different degrees of surface confinement and different magnitude of the pressure variations in the container inducing wall vibrations that act back on the bubble dynamics. The feedback from the liquid movement is expected to be weakest with free surface and strongest with deep immersion of the confining piston.

In our study, we vary the laser pulse energy to alter the maximum size and oscillation time of the bubble produced in the contain center. The stronger bubble expansion with increasing pulse energy results in larger bubbles and a stronger feedback from the bubble’s surroundings. Moreover, the relative phase between bubble oscillations and feedback-pressure waves changes. Altogether, this leads to a complex variation of bubble dynamics in dependence of pulse energy, or primary bubble size, respectively.

The bubble dynamics is recorded by high-speed photography and by simultaneous measurements of the intensity variations of a continuous wave probe laser beam yielding bubble oscillations times. Furthermore, we use a large-area pressure transducer as piston confining the liquid surface to be able to record the pressure evolution in the container. The combined results from all three approaches are evaluated to deduce the interaction mechanisms governing the bubble dynamics in semi-confined geometry. The analysis is supported by numerical simulations using the Rayleigh-Plesset model of bubble dynamics together with extra pressure terms describing the influence of the compressive pressure produced by liquid confinement as well as the pressure waves produced by the vibrations of the elastic container walls and the oscillations of the liquid mass in the cuvette corners.

The results of the present study are of interest for a better understanding of the mechanisms and side effects of intraluminal laser surgery and material processing in confined or partially confined geometry, which can guide parameter selection and process optimization. Biomedical examples are pulsed laser angioplasty in blood vessels [35], [36], and laser lithotripsy in the ureter [6], [37], [38], [39]. Furthermore, laser-induced bubble dynamics in semi-confined geometry plays a role in microfluidic applications [27], [40], [41], [42]. The results provide also a guideline, which conditions must be met to *avoid* unwanted feedback from the water cell on bubble oscillation in studies that aim at the investigation of spherical bubble dynamics in free liquid.

## 2 Experimental methods

### 2.1 Bubble generation

**Fig. 2:**
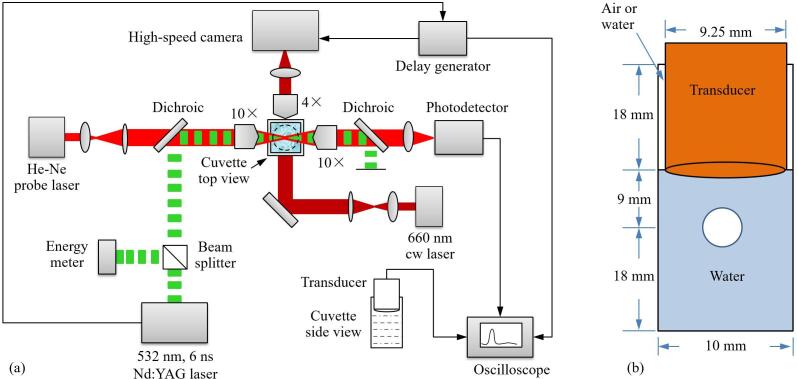
(a) Experimental arrangement for investigating the dynamics of the laser-induced bubble in a small container with elastic walls and different degrees of surface confinement. A spherical bubble is generated by focusing a Nd:YAG laser pulse onto the central axis of the cuvette. Bubble oscillations and pressure evolution are recorded simultaneously by high-speed photography, transmission measurement of a cw laser probe beam and detection of the far-field pressure amplitude with a piezoelectric transducer that also acts as piston confining the cuvette surface. The circumference of the transducer is shown as dashed line in the top view drawing. (b) Dimensions of cuvette and piston, and location of the laser-induced bubble.

Fig. 2(a) shows the experimental setup for laser-induced bubble generation using a frequency-doubled Q-switched Nd:YAG laser (Quantel Q-smart 450, 532 nm wavelength, 6 ns pulse duration, 10 Hz repetition frequency, and available pulse energies of up to 220 mJ). The laser pulses are first divided by a non-polarizing 10:90 plate beam splitter (Thorlabs, BSN10R). The weak reflected part is directed onto an energy meter (Ophir, PE50-DIF-C) for pulse energy measurement. The transmitted part is focused into a water-filled quartz cuvette (10 mm inner size, 45 mm height, and 1.2 mm wall thickness) using a long-distance microscope objective (Daheng optics, GCO-2131) with 10 × magnification, 0.25 numerical aperture, and 15 mm working distance. The pump laser beam has a diameter of 6.5 mm at the laser exit, a divergence angle of < 0.5 mrad (manufacturer data), and the path length up to the focusing objective is 125 cm. Assuming a divergence of 0.4 mrad, the beam diameter at the objective is 7.5 mm. The objective had a posterior pupil diameter of 10 mm, and the effective numerical aperture is, thus, *NA* = 0.185.

Often a water-immersion objective built into the wall of the water cell is used to achieve an aberration-free focus [43] but this approach cannot be utilized in this study because the isotropy of the cuvette response would be compromised if an objective was mounted in one of its walls. Therefore, we had to cope with the spherical aberrations that are introduced by focusing light through a plane cuvette wall using an objective corrected for free-space operation [44], [45]. We had to find a compromise between the prolongation of plasma length due to the strong increase of spherical aberrations with increasing *NA* [45], [46] and the decrease of the Rayleigh length of the diffraction-limited focus, which scales with 1/*NA*^2^. We tried objectives with *NA* = 0.25, 0.4 and 0.6 in conjunction with the unexpanded laser beam and found that the objective with *NA* = 0.25 produced the best results, with shortest plasma lengths and approximately spherical bubble dynamics.

Fig. 2(b) shows the dimensions of the cuvette and of the piston-like transducer, which partially confines the surface of the liquid in the cuvette. With weak confinement, 67.2 % of the surface area is covered but the liquid can still move freely in the corners of the cuvette. For stronger confinement, the water is filled up to the top of the cuvette. Here, the liquid movement is impeded by the large wall surface with no-slip boundary condition (zero velocity at the wall), which induces an additional friction compared to the case with weak confinement.

### 2.2 High-speed photography of bubble dynamics

To precisely monitor the temporal evolution of bubble dynamics, three methods are simultaneously employed, as depicted in Fig. 2(a): high-speed photography, transducer measurements of the acoustic transients, and detection of the light scattering by a probe laser beam.

A high-speed camera (Photron, Fastcam SA-Z) is used to record the cavitation bubble dynamics at framing rates of 210,000f/s (384 pixels × 160 pixels) and 480,000f/s (128 pixels × 80 pixels). The bubbles were imaged onto the CMOS camera chip (20 µm pixel size) with a combination of a 4×, NA = 0.1 microscope objective and a Navitar zoom lens. The zoom factor was adjusted such that the largest bubble images would fit into the image size on the sensor. This led to a magnification factor of 3.1, as determined by imaging a calibration scale onto the sensor with known pixel size.

For illumination, we used the continuous emission of a semiconductor laser (Coherent, OBIS660) at 660 nm wavelength and a beam expander (Daheng optics, GCO-2503). The exposure time is determined by electronic camera gating and was 160 ns for each frame. Because a cw semiconductor laser was used for illumination of the bubbles, the background in the bubble images exhibited speckles at ‘regular’ illumination intensity. To reduce the speckles, the camera was slightly overexposed in the bright background part of the images, while the bubble images remained dark. This isolates bubble images and facilitates the evaluation of bubble sizes. Since speckles remain in the transition zone between bright and dark image parts, the margin of the cavitation bubbles appears a little granulated, especially for small-sized bubbles and at the highest frame rate, where the pixel number per image is smallest.

Thresholding by moderate overexposure could, in principle, lead to a slight reduction of evaluated bubble sizes compared to regular thresholding at 50 % gray level (up to half of the width of the coherent edge function) but this effect was avoided by appropriate calibration of the *R*_max_ determination procedure. We determined the pixel number within the bubble outline to evaluate the bubble cross section and calculated the equivalent spherical radius from this value. Pixels at the bubble rim were attributed to bubble or background by setting a grey level threshold. This threshold was calibrated with bubbles in free liquid, where the relationship between measured oscillation times and *R*_max_ is theoretically known. The threshold was set in a way that *R*_max_ determined from the evaluation of the photographs agreed with the value determined from the bubble oscillation time *T*_osc_. The same threshold was then used also for images taken with surface confinement.

The spatial resolution is governed by the small number of pixels in each image. The camera’s pixel size is 20 µm. According to the Nyquist criterion, spatial periods down to 40 µm can then be resolved in image space, which with 3.1 × image magnification corresponds to 12.9 µm width of a just resolvable line pair in object space. Compared to this value, the measurement accuracy for *R*_max_ was largely improved by determining the pixel number within the bubble outline and calculating the equivalent spherical radius from this value. This procedure averaged over the pixelated outline of some bubble images and enabled a fairly precise determination of bubble radius in the investigated range 200 µm < *R*_max_ < 800 µm.

### 2.3 Acoustic and probe beam scattering measurements for determination of bubble oscillation times

A flat piezoelectric pressure transducer (Olympus, V324-N-SU) with a diameter of 9.25 mm and a central frequency of 25 MHz (14–––32 MHz bandwidth) is employed for far-field detection of the acoustic transients produced by the optical breakdown and bubble collapse. It serves, at the same time, as piston confining the surface of the water cuvette. The transducer is positioned approximately 9 mm above the laser focus, and different degrees of confinement are realized by different levels of the water surface relative to the sensor plane (Fig. 1).

The frequency response of the transducer in the MHz range is well suited for the detection of the time interval between the breakdown and collapse shock waves, which provides the bubble oscillation time *T*_osc_. However, the transducer is not sensitive enough for measuring wall-vibration-induced low-amplitude pressure variations in the kHz range.

A determination of *T*_osc_ from the transducer signal is not possible in experiments with free liquid surface. Therefore, a probe beam scattering technique is additionally implemented to measure oscillation times. A continuous probe beam emitted by a He-Ne laser (Thorlabs, HNL020RB) and expanded by a beam expander (Thorlabs, GBE05-A) is adjusted collinear with the pulsed laser beam and focused into the cuvette. The transmitted probe light is collimated by a microscope objective with the same numerical aperture as the focusing lens and focused on an AC-coupled amplified photoreceiver (FEMTO, 25 kHz-200 MHz bandwidth). To block the irradiation of the pump laser pulse, a long-pass dichroic mirror with a cut-on wavelength of 567 nm (Thorlabs, DMLP567R) and a notch filter with a central stop-band wavelength of 533 nm (Thorlabs, NF533-17) are placed in front of the photoreceiver. The bubble oscillations in the focal region and the shock wave emissions lead to fluctuations in the intensity of the transmitted probe beam, with peaks during breakdown and bubble collapse. This technique allows for the detection of nano-sized bubbles as small as 150 nm, which is far smaller than the optical diffraction limit [43]. The signals from photodetector and pressure transducer are both recorded using a digital oscilloscope (Rohde & Schwarz, PTE1204). The timings of pump laser emission, high-speed camera recording, and oscilloscope recordings are controlled and synchronized by a delay generator (Stanford Research Systems Inc., DG645).

### 2.4 Measurement series

The combination of high-speed photography, probe beam scattering technique and acoustic measurements enables an accurate and reliable single-shot determination of bubble dynamics and oscillation times, which, in turn, enables to collect a large data basis on the parameter dependence of bubble dynamics. This technique was used to explore the laser induced spherical bubble dynamics as a function of bubble size in the three settings with partial confinement shown in Fig. 1.

The height of the neck between transducer and cuvette wall is *h* = 18 mm. In order to bridge between the data sets obtained with the settings of Fig. 1(b) and 1(c), we performed additional measurements of bubble oscillation times, in which the height of the liquid in the neck between piston and cuvette walls was stepwise increased.

As a reference for the data obtained with small cuvette, we performed a series of experiments simulating bubble oscillations in free liquid. For this purpose, we used a larger glass cuvette (30 mm × 30 mm × 45 mm) with free surface, where the interior walls were covered with acoustically absorbing material. The bubble was produced 18 mm above the cuvette bottom as in the small cuvette, and the free surface had no influence on the bubble dynamics. Altogether, 320 radius-time curves were recorded photographically, and/or oscillation times of 5400 events were recorded by acoustic or probe beam measurements.

The bubble oscillation times *T*_osc1_ and *T*_osc2_ were determined by an automatic algorithm enabling to handle a large amount of data. When the breakdown and collapse signal peaks in the acoustic signal could be clearly identified, only this signal was used to determine *T*_osc1_ and *T*_osc2_. However, with partially confined geometries, the acoustic signal in the first or second collapse was sometimes weak and its identification was compromised by additional signals arising from secondary cavitation. In these situations, we used the additional information provided by the light scattering signal to narrow down the time window of bubble collapse, from which we then selected the acoustic peaks corresponding to the 1st and 2nd collapses.

## 3 Experimental results

### 3.1 Laser-induced cavitation in a large cuvette simulating free liquid

**Fig. 3:**
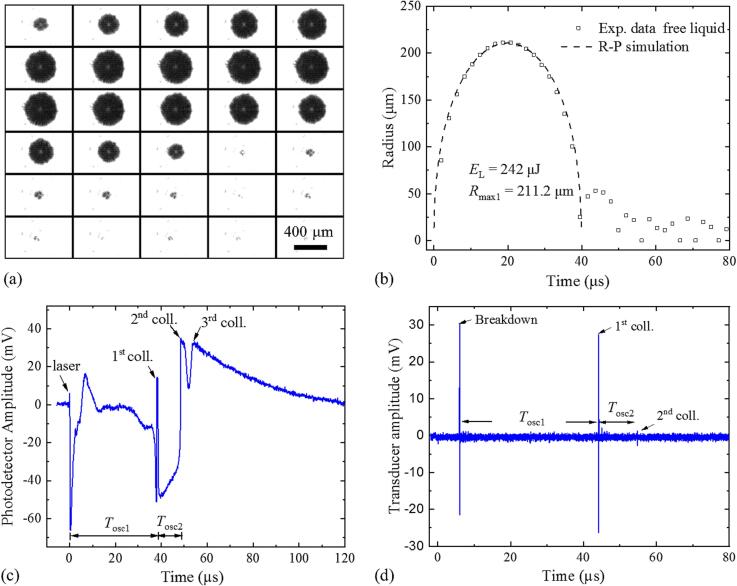
Cavitation bubble dynamics produced by a 242-μJ laser pulse in a large cuvette with free liquid surface and walls covered by acoustic absorbers. (a) Photographic series taken at 4.8 × 10^5^ frames per second with 2.1 µs interframing time. (b) Experimental *R*(*t*) data and R-P simulations of the first oscillation. (c) Probe beam transmission signal showing the first three bubble oscillations. (d) Far-field acoustic signal. Breakdown occurs at *t* = 0 but the signal is shifted by ∼ 6.0 μs due to the acoustic transit time from the laser focus to the transducer. The probe beam signal yields bubble oscillation times *T*_osc1_ = 38.24 μs and *T*_osc2_ = 10.40 μs, in good agreement with the results of the acoustic method, which are *T*_osc1_ = 38.12 μs and *T*_osc2_ = 10.60 μs, respectively. The corresponding maximum bubble radius is *R*_max1_ = 211.2 μm.

Fig. 3 presents the bubble dynamics in a large cuvette simulating free liquid. A photographic image series of a single cavitation event with *R*_max1_ = 211.2 μm that was produced at a pulse energy *E*_L_ = 242 µJ is shown in Fig. 3(a), and the temporal evolution of the bubble radius, *R*(*t*), is depicted in Fig. 3(b). It agrees well with simulations by the Rayleigh-Plesset (R-P) model, which are described in section 4 further below.

Fig. 3(c) displays the intensity changes of the probe beam induced by bubble oscillations. They are particularly strong immediately after breakdown and during the final stage of bubble collapse. Therefore, the oscillation times of the cavitation bubble can be precisely determined. Fig. 3(d) shows the corresponding transducer signal. The first signal peak denotes the breakdown shock wave, and the second peak and a small third peak represent the collapse shock waves emitted by the first and second collapse of the cavitation bubble, respectively. The shock wave signals from optical breakdown and bubble collapse are bipolar, similar to the signals in Ref. [47], although in reality monopolar compressive transients are emitted, which are followed by a long rarefaction wave during the subsequent bubble oscillation. The bipolar shape is an artifact because with long cable and 50 Ω input-resistance at the oscilloscope, the transducer signal is the derivative of the original signal, which makes the trailing edge of the pressure transient appear as negative signal [48], [49].

The bubble shape in Fig. 3(a) is largely spherical during the first and subsequent oscillations, with some irregularities after the first collapse. In free liquid, the pressure transient emitted at spherical bubble collapse has mostly a similar or even higher amplitude than the breakdown transient [2], [50] but it is slightly smaller in Fig. 3(d). This may be caused by deviations from spherical symmetry due to the elongated plasma shape that also cause the shape irregularities in the second oscillation.

### 3.2 Bubble dynamics in a small cuvette with different degrees of surface confinement

**Fig. 4:**
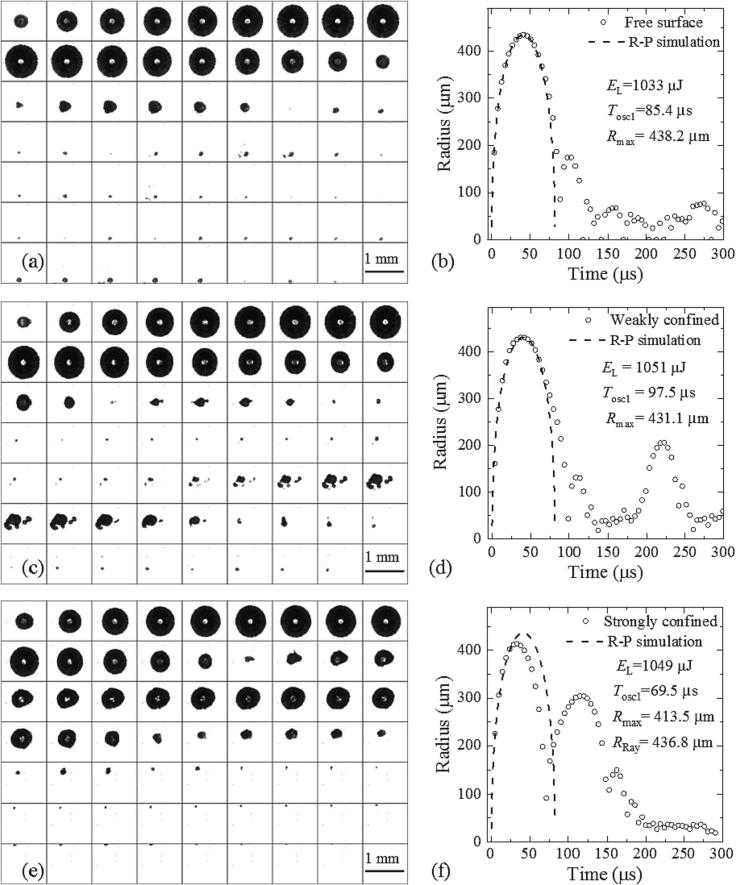
Bubble dynamics in a small cuvette with elastic walls for various surface confinement conditions at approximately equal pulse energies. (a), (b) free surface. (c), (d) weakly confined surface, (e), (f) strongly confined surface. The high-speed photographic series in (a), (c), and (e) were taken at 2.1 × 10^5^ frames/s with 4.76 μs interframing time. The experimental data points in (b), (d), and (f) correspond to equivalent spherical radius values, when the bubble deviates from spherical shape. For free surface and weak confinement, R-P simulations were performed to match the measured *R*_max_ values, whereas for strong confinement, they match the *R*_max_ value corresponding to the measured oscillation time in free liquid at *E*_L_ = 1049 µJ. This value is denoted *R*_Ray_.

Fig. 4 shows the bubble dynamics in a small cuvette with elastic walls for the three cases shown in Fig. 1. The laser pulse energies (*E*_L_ ≈ 1 mJ) and maximum bubble sizes (*R*_max1_ ≈ 430 µm) are similar in all three cases but the collapse dynamics and subsequent oscillations exhibit marked differences from the dynamics in free liquid and among each other.

With *free surface* (Fig. 4b), the time period of the first bubble oscillation is slightly prolonged compared to the dynamics in free liquid represented by the R-P curve. The second oscillation is larger than for free liquid, which becomes obvious by comparison with Fig. 3(b). The prolongation of the first oscillation is more pronounced with *weak surface confinement* (Fig. 4d). Here a pronounced re-oscillation appears at *t* ≈ 200 µs, about 100 µs after the end of the first oscillation and 70 µs after the end of the second oscillation. With *strong confinement*, both the first oscillation time and maximum bubble radius are compressed but the amplitude of the second oscillation is much larger than in free liquid.

The photographic image series show that the bubble remains approximately spherical during the first oscillation in all three cases. However, the overall bubble movement reflects the presence of a free surface in Fig. 4(a) and the partial confinement by a solid boundary in Fig. 4 (c) and (e). The free surface causes a downward movement of the bubble [11], whereas the solid boundary immersed into the liquid from the top results in an upward movement [11], [12] because it is closer to the bubble than the solid bottom of the cuvette.

The prolongation of the first oscillation, the enlargement of the second oscillation and the re-oscillation in Fig. 4(c), (d) are attributed to the bubble-induced movement of the cuvette walls that with a certain delay causes underpressure or tensile stress in the bubble’s surrounding. The re-oscillation starting at *t* ≈ 200 µs is ascribed to the wall-induced tensile stress wave rather than to high internal bubble pressure as no extra laser energy is deposited. This interpretation is supported by the observation that the re-expansion starts from small seed bubbles, which also explains the irregular shape of the re-expanded bubble cluster.

When the space in the corners of the cuvette around the piston is filled with liquid, the bubble movement is more strongly confined than when the piston just touches the liquid surface and the liquid can move freely in the corners. As visible in Fig. 4(e) and (f), the stronger confinement causes a reduction of the amplitude and period of the first oscillation compared to free liquid. The reduction of *T*_osc1_ is followed by a prolongation of *T*_osc2_ caused by the feedback from the bubble-induced cuvette wall oscillation.

**Fig. 5:**
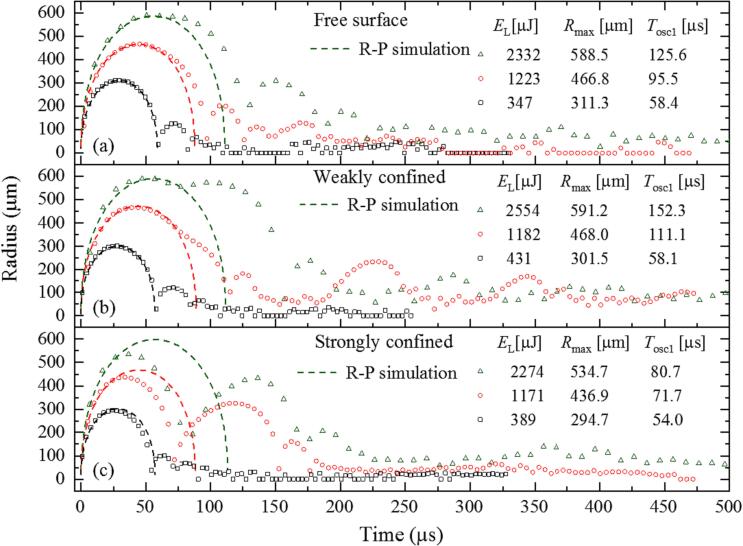
Radius-time curves of bubbles with various size for (a) free liquid surface, (b) weakly confined surface, (c) strong confinement.

These features become even more obvious in Fig. 5, which shows the dynamics of bubbles of different size. With free and weakly confined surface, *T*_osc1_ is most strongly prolonged for the largest bubble, whereas with strong confinement, it is here most strongly reduced. In Fig. 5(c), the combination of initial compression followed by rarefaction leads for the largest bubble (*E*_L_ = 1171 µJ) to the intriguing observation that *T*_osc2_ = 80.0 µs is even longer than *T*_osc1_ = 71.7 µs. Fig. 5(b), shows that with weak confinement several delayed re-oscillations can follow the first and second oscillation. Because of the 200-µs delay, it is unlikely that the re-oscillations are due to the initial cuvette wall vibrations which prolong the first and/or second oscillations. We attribute the re-oscillations to feedback from the liquid movement in the cuvette corners.

**Fig. 6:**
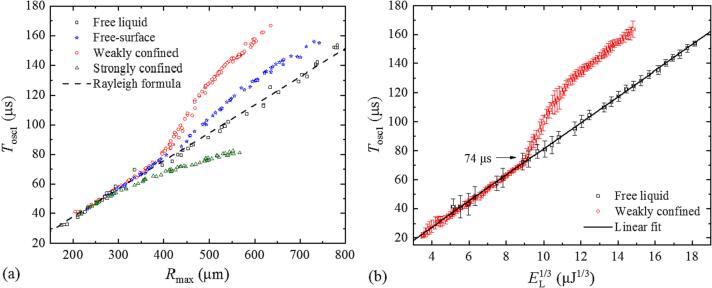
(a) Bubble size dependence of first oscillation time, *T*_osc1_ (*R*_max_), under various surface conditions and in free liquid. The data were evaluated from 320 high-speed photographic image series. (b) Oscillation time *T*_osc1_ as a function of *E*_L_^1/3^ for free liquid and weak surface confinement. The data for weakly confined liquid surface are obtained from probe beam scattering and transducer measurements at pulse energies in the range between 40 µJ and 3.3 mJ. Each data point is an average over 20 measurements, and 1380 measurements were analyzed in total. We plot *T*_osc1_ as a function of *E*_L_^1/3^ rather than *R*_max_ because *R*_max_ is not directly available from the measurement. However, well above breakdown threshold, most laser energy is absorbed, and the bubble volume is approximately proportional to *E*_L_. The bubble radius, in turn, is approximately proportional to *E*_L_^1/3^.

Fig. 6 shows the changes of *T*_osc1_ as a function of bubble size in more detail. Here, also data for free liquid are included as reference for the other cases. Interestingly, for strong confinement *T*_osc1_ decreases gradually with increasing *R*_max1_, whereas the increase of *T*_osc1_ for the cases with free liquid surface and weak confinement exhibits a threshold behavior. In Fig. 6(a), where data are evaluated from high-speed photographic series, one sees an increase of *T*_osc1_ relative to the oscillation time in free liquid somewhere between *T*_osc1_ = 70 µs and *T*_osc1_ = 80 µs. In Fig. 6(b), where a larger data base from probe beam scattering and acoustic beam measurements is incorporated, one can identify *T*_osc1_ = 74 µs as threshold for the oscillation time prolongation. The bubble size above which the collapse phase is prolonged is *R*_max_ ≥ 390 µm, as seen in Fig. 6(a).

We have seen in Fig. 5 that the relation between first and second oscillation period changes in a complex fashion with increasing bubble size and confinement. This relation is explored in more detail in the next section.

### 3.3 Relationship between second and first bubble oscillation time

**Fig. 7:**
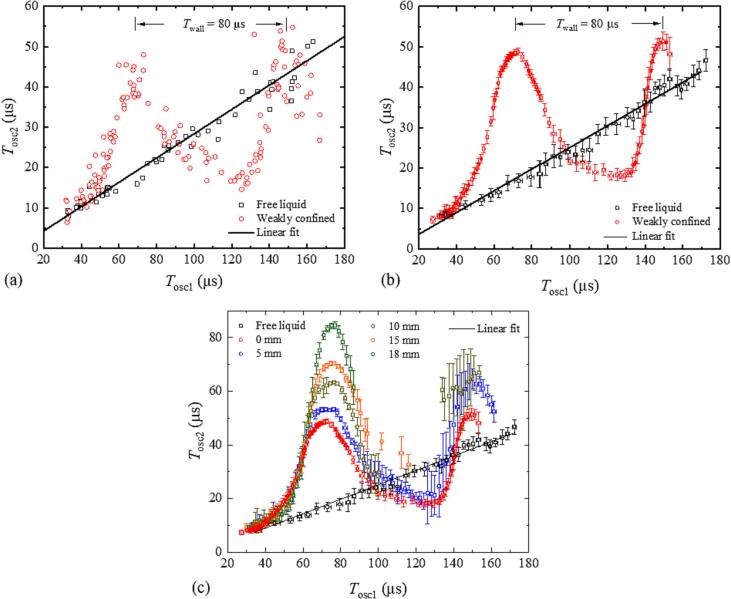
Dependence of the second bubble oscillation time on the first oscillation time, *T*_osc2_ (*T*_osc1_). In (a), the data are determined from high-speed photographic image series and in (b) from probe beam scattering and transducer measurements. The ratio *T*_osc2_ /*T*_osc1_ is constant for free liquid but exhibits a modulation with weakly confined liquid surface. The modulation period is 80 μs. (c) shows *T*_osc2_ (*T*_osc1_) for different liquid heights *h* in the cuvette corners. Each data point is an average over 20 measurements in (b) and over more than 10 measurements in (c).

Fig. 7 shows *T*_osc2_ (*T*_osc1_) for different liquid heights *h* in the cuvette corners in comparison with free liquid. Here, the period of the first oscillation represents the bubble size because with surface confinement we cannot directly plot *T*_osc2_ (*R*_max1_) due to the complex relationship between *T*_osc1_ and *R*_max1_ that we have seen in Fig. 5, Fig. 6.

The ratio *T*_osc2_ /*T*_osc1_ varies with bubble size – with increasing *T*_osc1_ it first becomes larger than the value in free liquid, then smaller, and then larger again. We attribute this behavior to the pressure variations from the bubble-induced cuvette wall vibrations. The bubble expansion induces an outward movement of the cuvette walls that results in a tensile wave in the liquid, when the bubble starts to collapse while the cuvette wall continues to move outward. The tensile wave is followed by a compressive wave, when the elastic cuvette wall swings back and then again by a tensile wave when it swings out again. The modulation of *T*_osc2_ /*T*_osc1_ is linked to the relative phase between the bubble oscillation, which varies with bubble size, and the wall oscillations that remain constant. The 80-µs spacing of the *T*_osc2_ /*T*_osc1_ maxima indicates the eigenperiod *T*_wall_ of the wall oscillations.

We see in Fig. 7(c) that *T*_wall_ remains approximately constant, when the liquid height in the cuvette corners is increased but the amplitude of the *T*_osc2_ /*T*_osc1_ oscillations increases with *h*, and the peak of the first oscillation slightly shifts to larger *T*_osc1_ values. The second peak of the oscillation is not visible for *h* ≥ 15 mm because for a given laser pulse energy *T*_osc1_ decreases with increasing confinement [Fig. 6(a)]. Since *E*_L_ is limited in the experiments, the *T*_osc2_ /*T*_osc1_ data cover only the range up to *T*_osc1_ ≈ 110 µs for *h* = 15 mm and up to *T*_osc1_ ≈ 90 µs for *h* = 18 mm.

The approximately constant period of the *T*_osc2_ /*T*_osc1_ oscillations indicates that they cannot just be linked to the variation of liquid mass in the cuvette corners in analogy with a Helmholtz resonator. In a Helmholtz resonator, the resonance frequency should change with different mass of the fluid in the neck around the transducer [51], [52], which is not the case in our experiments. The constant oscillation period can be explained if the vibration of the entire liquid container is considered rather than the oscillation of the liquid mass in the neck. The total liquid volume (*V*_maincuvette_ + *V*_neck_) changes little by adding some water in the neck. The main resonance in, for example, a wine glass is determined by the entire liquid volume *V*, and its frequency changes only slowly with *V* [53]. The constancy of the period of *T*_osc2_/*T*_osc1_ oscillations with varying *h* suggests that always the main mode is excited and higher modes play little role. These observations indicate that neither the analogy with a Helmholtz resonator nor different vibration modes of the container can explain the observed changes with *h*. However, they can be explained by an increasing confinement of the liquid movement. In Fig. 6(a), we see a strong reduction of *T*_osc1_, when the gap between transducer and cuvette wall is filled, and when laser-induced bubble is sufficiently large. In addition, the increase of the *T*_osc2_/*T*_osc1_ ratio with *h* observed in Fig. 7(c) is also linked to the decrease of *T*_osc1_, in combination with an increase of *T*_osc2_ that is visible in Fig. 5.

## 4 Theoretical model

The bubble dynamics investigated in this paper involves a complex interplay among the laser-induced primary bubble oscillations, which are influenced by the confinement of the liquid surface, the bubble-induced elastic wall vibrations, and the movement of the liquid in the cuvette corners. We pursue a modular approach to describe individual components of the interplay but do not attempt to integrate them into a unified comprehensive model. We use the Rayleigh-Plesset (R-P) model extended by an extra pressure term describing the bubble-induced external pressure *p*_e_(*t*) and present different forms of the term *p*_e_(*t*) that cover the three major mechanisms acting back on bubble dynamics: confinement of the liquid volume, cuvette wall oscillation, and liquid movement in the cuvette corners. In section 5, we will then compare experimental results under different confinement conditions to simulation results obtained by that form of the extended R-P model, which is most appropriate for the respective condition.

### 4.1 General form of the extended Rayleigh-Plesset model

The Rayleigh-Plesset equation of motion for a spherical bubble oscillator in an incompressible liquid with external pressure field reads [1], [54]

(1) $$\rho R\ddot{R}+\frac{3}{2}\rho {\dot{R}}^{2}=p_{\text{gn}}{\left(\frac{R_{\text{n}}}{R}\right)}^{3\kappa }+p_{\text{v}}-p_{\infty }-\frac{2\sigma }{R}-\frac{4\mu }{R}\dot{R}-p_{\text{e}}\left(t\right)$$

with

(2) $$p_{\text{gn}}=\frac{2\sigma }{R_{\text{n}}}+p_{\infty }-p_{\text{v}}.$$

Here, *R* denotes bubble radius and an overdot means differentiation with respect to time *t*. Other parameters are *ρ* the liquid mass density, *p*_v_ the saturated vapor pressure, *p*_∞_ the static pressure, *σ* the surface tension of the liquid, and *μ* the (dynamic) viscosity of the liquid. For room temperature (20 °C) and ambient pressure of 100 kPa, the following values are used in all simulations: *p*_v_ = 2330 Pa, *ρ* = 998 kg/m^3^, *σ* = 0.073 N/m, *p*_∞_ = 0.1 MPa, and *μ* = 10^-3^ Pa·s.

The bubble’s interior is treated as a non-condensable gas, and its time-varying gas pressure is described by the first term on the right side of Eq. (1), which utilizes the ideal gas law under adiabatic conditions. The parameter *R*_n_ denotes the equilibrium radius of the bubble at which the bubble pressure balances the hydrostatic pressure, *p*_gn_, denotes the pressure of the non-condensable gas inside the bubble at rest, and *κ* is the adiabatic exponent (the ratio of the specific heats at constant pressure and volume). For triatomic gas, *κ* = 4/3 [1].

The first two terms on the right side of Eq. (1) describe the internal pressure inside the bubble and the remaining terms denote the pressure outside the bubble. The last term, *p*_e_(*t*), refers to a time-varying component from an additional sound field. In our investigations, it describes the feedback from the cuvette walls and the liquid surface with weak and strong confinement. For a free liquid, *p*_e_ = 0.

For bubbles with *R*_max_ ≥ 200 µm that are investigated in the present paper, the influence of viscosity and surface tension can be neglected, and the bubble oscillation time in free liquid exhibits a linear relationship with the maximum bubble radius [55]

(3) $$R_{\max}=R_{\text{Ray}}=\frac{T_{\text{osc}}}{1.83}\sqrt{\frac{p_{\infty }-p_{\text{v}}}{\rho }}.$$

In the presence of an external pressure field, the Rayleigh-relation (3) does not apply, and *R*_max_ ≠ *R*_Ray_.

For simulations of laser-induced bubble dynamics, *R*_0_ should match the equivalent spherical plasma radius if the plasma volume is known from photographs, and *R*_n_ can then be used as fitting parameter to adjust the predicted bubble size to the experimentally measured oscillation time [2]. In the present experiments, the plasma size could not be determined from the high-speed photographic series because of their limited spatial resolution. However, it is known that well above bubble threshold (i.e. for the investigated bubble sizes *R*_max_ ≥ 200 µm) the plasma energy density is approximately constant [56]. In this regime, most of the incoming laser energy is absorbed, and the plasma volume increases approximately proportional to the pulse energy. Constant plasma energy density corresponds to a constant plasma pressure, which is according to Eq. (1) represented by the term (*R*_n_/*R*_0_)^3κ^. Therefore, we keep *R*_n_/*R*_0_ constant during the fitting procedure and tune *R*_0_ until the simulated *R*_max_ matches the experimentally determined value. We use a value *R*_n_/*R*_0_ = 10.4 adopted from Ref. [2], where it had been experimentally determined from i) measurements of plasma volume deduced from photographs of plasma luminescence and ii) transmission measurements of the incident laser light.

It should be noted that the above fitting procedure only works for the first bubble oscillation. The amplitude of the second oscillation is influenced by the energy loss through shock wave emission in collapse [2], which is not covered by the Rayleigh model but requires a more complex approach considering liquid compressibility.

### 4.2 Pressure evolving during confined bubble oscillations

When a laser-induced bubble is created in a fully confined liquid volume with rigid walls, the pressure around the bubble increases strongly during bubble expansion. For a compressible liquid with finite volume *V*, the volume change induced by the bubble oscillations is given by Δ*V* = −4π*R*^3^(*t*)/3, when the initial bubble size is neglected. The corresponding pressure change is Δ*P* = −*K*_1_(Δ*V*/*V*), where *K*_l_ is the bulk modulus of the liquid. Thus, the extra pressure *p*_e_(*t*) of Eq. (1) is here induced by the bubble oscillations and given by

(4) $$P_{\text{comp}}=K_{\text{l}}\frac{4\pi R^{3}\left(t\right)}{3V}.$$

The additional pressure arising from the liquid compression can be understood as elevated ambient pressure, which reaches its maximum value at *R*_max_, when the volume change is the largest. This interpretation assumes a quasi-instantaneous response to the bubble dynamics, which is justified even for a compressible liquid, if the sound transit time within the container is much smaller than the bubble oscillation time. The bubble-induced slow rise of ambient pressure in confined environment differs from the transient pressure rise upon optical breakdown in an infinite compressible liquid, where only liquid inertia counteracts the bubble expansion and a pressure transient (shock wave) is emitted into the surrounding liquid [2].

For water with bulk modulus of ∼2.18 × 10^9^ Pa, a 0.1 % change in liquid volume causes a pressure change of ≈2.2 MPa. Thus, bubble expansion in a fully confined finite water volume is counteracted by a strong pressure elevation in the liquid, which reduces *R*_max_. When container walls are elastically compressible but do not bend, the pressure rise is diminished by the elastic wall deformation. In this case, the effective total bulk modulus can be described by

(5) $$K_{\text{eff}}=\frac{K_{\text{c}}K_{\text{l}}}{K_{\text{c}}+K_{\text{l}}},$$

where *K*_c_ denotes the bulk modulus of the confining container [33], and *K*_1_ in Eq. (4) must be replaced by *K*_eff_ to calculate *P*_comp_.

With partial confinement, the pressure rise is lower than described by Eq. (4) for full confinement through rigid walls. However, the confinement conditions consisting of the quartz cuvette walls, the transducer piston, and the water columns in the cuvette corners are very complex. We are unable to describe it by an effective bulk modulus *K*_eff_ with explicit consideration of the constituting bulk modulus terms, and the response of the liquid in the cuvette corners cannot at all be characterized by an elastic bulk modulus. Therefore, as a first order approximation, we introduce a fitting parameter *C* as proportionality factor to describe the overall change of *P*_comp_ with bubble volume 4/3π*R*^3^.

The pressure change in liquid can then be described by

(6) $$P_{\text{comp}}=C\;K_{\text{l}}\frac{4\pi R^{3}\left(t\right)}{3V}.$$

With free liquid, *C* = 0; for complete confinement with rigid walls, *C* = 1, and for intermediate degrees of confinement, 0 < C < 1. We will see in section 5.1 that for strong confinement a single value *C* = 0.45 provides a good fit between measured bubble size and simulations in the whole investigated *R*_max_ range, when *P*_comp_(*t*) from Eq. (6) is used as external pressure term in Eq. (1). For free surface in a small container and weak confinement, no change of the bubble expansion phase and *R*_max_ compared to free liquid was observed within the measurement accuracy. Therefore, we set *C* = 0 in the simulations of bubble oscillations under weak confinement although it may actually be slightly larger than zero.

### 4.3 Feedback on bubble oscillations through vibrations of the elastic cuvette walls

**Fig. 8:**
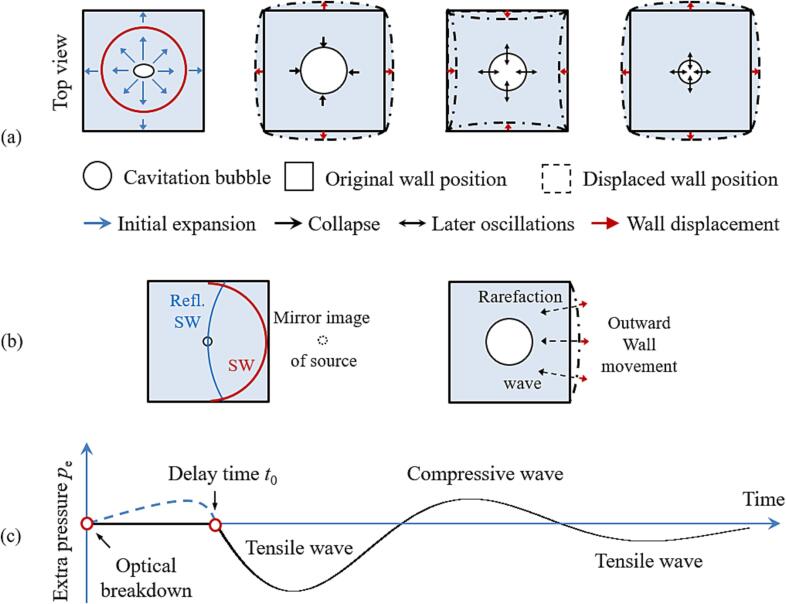
(a) Generation of additional pressure in partially confined liquid through elastic wall vibrations generated by momentum transfer from laser-induced shock wave and bubble expansion. (b) Divergence of the breakdown shock wave after the reflection at the cuvette wall in comparison with the weak focusing of the rarefaction wave induced by the outward movement of the wall. (c) Pressure wave induced by the wall vibrations shown in (a) that acts back on the bubble.

The generation of laser bubbles in a glass cuvette by millijoule laser pulses goes along with a sharp ringing noise lying in the audible frequency range. This sound cannot come from the laser-induced shock waves with non-audible frequencies well above 1 MHz but arises from the shock-wave- and bubble-induced vibrations of the wall, which are illustrated in Fig. 8(a). The pitch of this sound depends on the size of the cuvette, with larger containers producing lower frequencies. In the present investigations, the pitch was towards the upper end of audible frequencies. This is consistent with the eigenperiod *T*_wall_ = 80 µs of wall oscillations (corresponding to a frequency of 12.5 kHz) that was deduced in section 3.3 from the oscillations of the *T*_osc2_ (*T*_osc1_) curve. For comparison: the resonant frequency of a liquid-filled wine glass is in the order of 1–1.5 kHz [6], and one would expect from a small cuvette with 10 × 10 mm^2^ cross section to have a considerably higher resonance frequency, in agreement with the experimental observations.

Hence, the cuvette walls do not only confine the liquid volume but their breakdown-induced vibrations also produce a sequence of negative and positive pressure changes that can influence the bubble dynamics. The vibrations are the combined effect of the momentum transfer from the impacting breakdown shock wave and of a longer-lasting pressure rise caused by the bubble expansion.

The relative importance of both effects can be assessed by comparing the magnitude of bubble energy and shock wave energy acting on the wall. The breakdown shock wave is strongly damped in the immediate vicinity of the plasma and loses more than 80 % of its initial energy during the 5 mm propagation to the wall [2], [3], [35], [57]. By contrast, the full bubble energy can act on its surroundings, because viscosity effects are negligible for millimeter-sized bubbles in water. The energy of bubbles produced by millijoule laser pulses was found to be 3–4 times larger than the shock wave energy at 10 mm distance from the plasma [35], [57]. It is still 2–3 times larger at a distance of 5 mm, which is the propagation distance to the cuvette wall in our experiments. The comparison suggests that the action of the bubble on the container wall is larger than that of the shock wave although the shock wave contribution is significant.

A direct feedback of the reflected shock wave on the bubble oscillations is unlikely because only 10–15 % of the shock wave energy arrive at a distance of 10 mm [57], [58], and the shock wave continues to diverge after reflection, as illustrated in Fig. 8(b). By contrast, the rarefaction wave produced by the outward motion of the cuvette wall is slightly focused towards the bubble. Since the traveling time to the wall and back to the bubble is ≤ 6 µs, the impulsive shock pressure should affect mostly the early bubble expansion phase. However, no change of the bubble expansion dynamics compared to free liquid is observed in any of our experiments with the small cuvette; detectable changes start only in the collapse phase. We conclude that both the impulsive acceleration by the shock wave and the longer-lasting acceleration by the bubble expansion act indirectly back on the bubble, mediated through the cuvette wall oscillations. The oscillations induce a pressure wave around the bubble that is depicted in Fig. 8(c).

The bubble expansion produces an elevated pressure in the liquid [indicated by the dashed line in Fig. 8(c)], which causes the centers of each cuvette wall to move outward. The outward movement continues because of inertia even when the bubble in the cuvette center starts to collapse. This causes an underpressure or even tensile stress in the bubble’s surrounding, which prolongs its collapse, as observed in Fig. 4, Fig. 5. The rarefaction wave is followed by compressive stress, when the cuvette walls swing back, followed by another rarefaction wave, when it swings out again. The wall oscillation is damped because it was excited by the first bubble expansion, and the bubble’s action on the wall ceases with decreasing amplitude of the bubble oscillations. The relative phase between wall and bubble oscillations at later times depends on bubble size.

We describe the additional pressure acting on the bubble because of the wall vibrations by a sinusoidal function with exponential damping term:

(7) $$P_{\text{vib}}\left(t\right)=\left\{\begin{array}{cc} 0, & \text{if}\;t⩽t_{0}\\ -A\left(\gamma \right)e^{-\left(t-t_{0}\right)/\tau }\sin\left[\frac{2\pi }{T_{\text{wall}}}\left(t-t_{0}\right)\right], & \text{if}\;t>t_{0} \end{array}\right),$$

(8) $$\mathrm{with}\;\gamma =R_{\max}/L\;and\;A=\alpha \;\gamma .$$

Here, *γ* represents the dimensionless bubble size in relation to the inner length *L* of the cuvette. The vibrational pressure, *P*_vib_, is described by a damped sine function with period *T*_wall_ and an amplitude *A.* The damping term is an exponential decay function with a decay time *τ*. The pressure amplitude *A* depends on the dimensionless bubble size *γ* = *R*_max_/*L*. As a first-order approximation, we assume *A* = α *γ*, where *α* is a constant pressure amplitude coefficient linking *A* to the dimensionless parameter *γ.*

It is worth noting that the strength of the vibrational pressure at the cuvette center is much greater than the amplitude of the pressure *P*_comp_, which initiates the vibrations. The pressure elevation arising from the bubble expansion in confined liquid simultaneously pushes on all surrounding walls, leading to synchronous vibrations, while the resulting vibrational pressure waves are focused towards the cuvette center, where the bubble is located. The amplitude of *P*_vib_ increases with increasing confinement and can, for large *R*_max_, even reach negative values sufficient to induce secondary cavitation bubbles [Fig. 4(c)]. The values of *α* and *τ* are used as fitting parameters.

The vibrational oscillation time *T*_wall_ corresponds to the wall’s eigenperiod and is, therefore, fixed. It is set to 80 μs, according to the modulation of the *T*_osc2_ /*T*_osc1_ curve in Fig. 7. The time delay *t*_0_ between optical breakdown and the onset of the feedback of the wall vibrations on bubble oscillations depends on laser pulse energy and the degree of confinement. Large bubbles under strong confinement excite the wall oscillation earlier but their collapse sets in later at a time, which depends on the degree of confinement. Therefore, *t*_0_ varies from case to case and is also used as fitting parameter.

### 4.4 Pressure arising from the liquid movement in the cuvette corners

We attribute the re-oscillations of mid-sized cavitation bubble that set in about 200 µs after optical breakdown [Fig. 4(d) and 5(b)] to the bubble-induced liquid movement in the cuvette corners. When the cuvette is filled to the top, the movement is strongly inhibited by viscous damping in the 18 mm high boundary layer. When the transducer just touches the liquid surface, some friction may still arise from flow components parallel to the transducer bottom and cuvette walls but it is much weaker than in the strong confinement case, and the liquid mass oscillation can get a larger momentum.

The additional pressure caused by the liquid movement has similar characteristics as the pressure wave produced by the wall oscillations: the liquid is pushed upward by the expansion of the primary bubble, continues to move due to its inertia, and moves back once the kinetic energy of the flow has been fully converted to potential energy of an elevated liquid column. The inertial upward motion causes underpressure around the collapsing bubble, and the downward motion induces an overpressure. The quadrupole geometry of pressure sources in the cuvette corners leads to a focusing of pressure variations on the central cuvette axis. Moreover, the pressure variations in the cuvette will excite wall vibrations, as described in the previous section.

Since a detailed description of the spatiotemporal pressure evolution would be very complex, we use the similar approach as presented in the previous section also for simulating the pressure variations by the movement of the liquid surface. We take the starting times *t*_start_ of the re-oscillations from the measured *R*(*t*) curves and use *A*, *τ* and the period of the liquid mass oscillation, *T*_osc,lm_, as fitting parameters. With these changes, Eq. (7) becomes

(9) $$P_{\text{osc,lm}}\left(t\right)=\left\{\begin{array}{c} \;\;\;\;\;\;\;\;\;\;0,\;\;\;\;\;\;\;\;\;\;\;\;\;\;\;\;\;\;\text{if}\;t⩽t_{\text{start}}\\ -A\left(\gamma \right)e^{-\left(t-t_{\text{start}}\right)/\tau_{\;}}\sin\left[\frac{2\pi }{T_{\text{osc,lm}}}\left(t-t_{\text{start}}\right)\right],\;\;\text{if}\;t>t_{\text{start}} \end{array}\right)$$

## 5 Simulations, and comparison to experimental results

### 5.1 First bubble oscillation under strong confinement

The elevated pressure produced by bubble expansion under strong confinement leads to a considerable reduction of *T*_osc1_, and the rarefaction wave resulting from the outward wall movement has apparently only a weak influence on the first oscillation although it may prolong *T*_osc2_. Therefore, we considered only *P*_comp_ as described by Eq. (6) for simulating the bubble dynamics during the first oscillation.

**Fig. 9:**
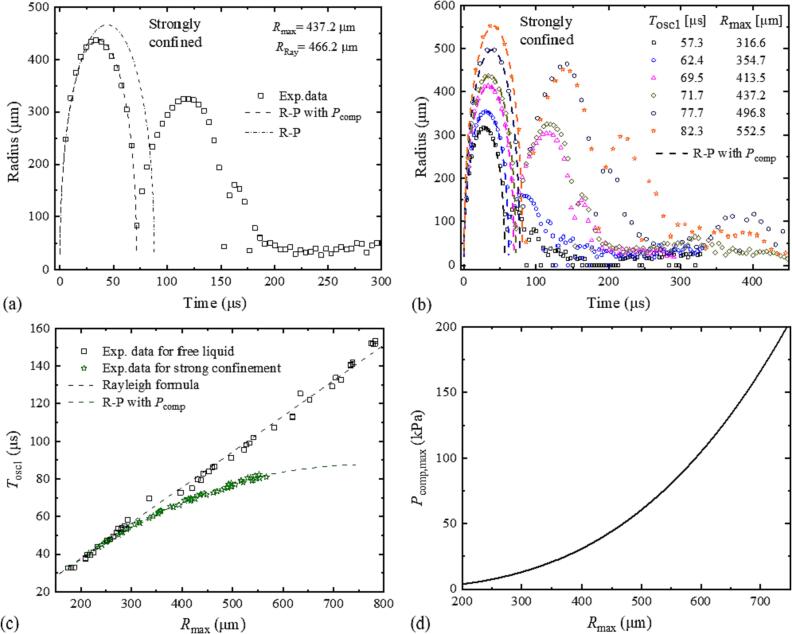
(a), (b) Simulations of the first bubble oscillation under strong confinement in comparison with experimental data. The simulations are based on the Rayleigh-Plesset model extended by *P*_comp_ [Eq. (6)]. In (a), also R-P simulation results for free liquid are given for comparison. (c) *T*_osc1_(*R*_max_) for free liquid and strong confinement. (d) *P*_comp_ at *R*_max_ plotted as a function of *R*_max_.

The volume of the liquid in the small cuvette up to the transducer surface is *V* = 2.7 ml, and the bulk modulus of water is *K*_l_ = 2.18 × 10^9^ Pa. The parameter *C* was tuned from 0.1 to 1 with a step size of 0.01 to find the best fit between numerical simulations and experimental data using a least-squares fitting scheme. A total of 65 radius-time curves were evaluated, and the best fits were achieved with *C* = 0.45. The simulation results in comparison with experimental data are presented in Fig. 9. With R-P extended by *P*_comp_, we find a very good agreement for the *R*(*t*) curves during the first oscillation [Fig. 9(a) and (b)] as well as for the *T*_osc1_(*R*_max_) curves [Fig. 9(c)].

A deviation from R-P dynamics in free liquid becomes prominent only at the late expansion phase although an elevated pressure is already generated during bubble expansion. This is because the strength of *P*_comp_ increases cubically with bubble radius. Therefore, it is relatively weak during the early expansion stage of the bubble and affects mainly the collapse phase. As a consequence, *T*_osc1_ is more strongly reduced by the extra compression pressure than *R*_max_. This explains why the *T*_osc1_(*R*_max_) curve in Fig. 9(c) lies below the straight line representing the Rayleigh relationship for free liquid.

At the present stage, we can only give a tentative explanation for the strong increase of confinement by adding 18 mm liquid height in the cuvette corners. It may be related to friction at the surfaces in the neck together with the impairment of the flow entering the neck that is caused by the piston edge. Details of the changes in flow velocity and pressure fields could be revealed by 3-D volume of fluid modeling but that goes beyond the scope of this paper.

The possibility to neglect *P*_vib_ in simulations of the first bubble oscillation for strong confinement does not mean that the vibrational pressure is weak. On the contrary, *P*_vib_ is here expected to be stronger than for weak confinement or free surface. However, because of the late onset of the vibrational pressure wave, it influences mainly the rebound of the collapsed bubble and leads to a significant increase of *R*_max2_ and *T*_osc2_. Unfortunately, the rebound cannot be modeled by the incompressible R-P model, which does not consider the energy dissipation by shock wave emission upon collapse that influences the rebound amplitude.

Fig. 9(d) presents the peak amplitude of *P*_comp_ at maximum bubble radius as a function of *R*_max_. It rises from approximately 4 kPa at *R*_max_ = 200 μm to 100 kPa at *R*_max_ = 600 μm. These values are consistent with hydrophone measurements by Orimi et al. [34]. They observed a peak pressure amplitude of 50 kPa for a sealed volume of 1.9 ml and maximum bubble sizes of 500 μm to 600 μm. The pressure increased to 150 kPa, when the liquid volume was reduced to 0.3 ml.

### 5.2 First bubble oscillation under weak confinement

With free surface and weak confinement, the bubble shows a prolonged collapse in the first oscillation, especially for large bubbles. Because a shortening of *T*_osc1_ is never observed, the compression pressure *P*_comp_, which would shorten *T*_osc__1_, is neglected in the simulations for free surface and weak confinement, and only the feedback from the cuvette walls through *P*_vib_ is considered for modeling the first bubble oscillation. The feedback from the liquid movement in the cuvette corners that sets in after a longer time delay will be discussed in the next section.

**Fig. 10:**
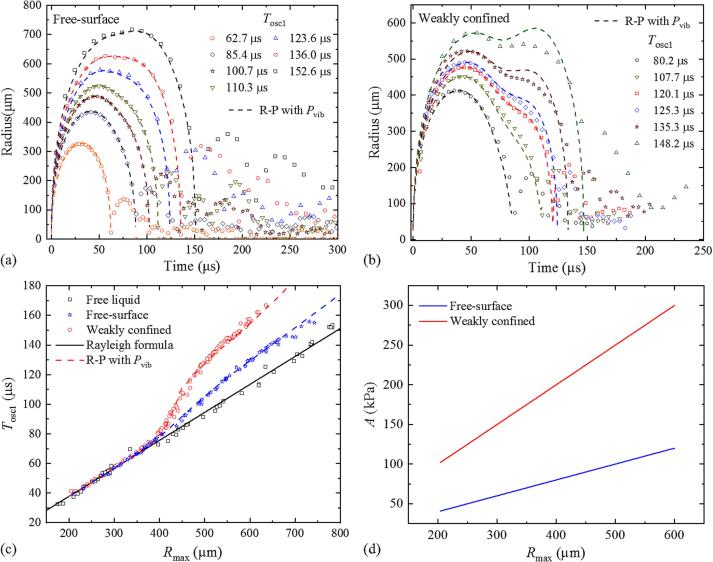
Simulation of the first bubble oscillation with free surface (a) and with weak confinement (b), in comparison with experimental data. Simulations are based on the Rayleigh-Plesset model extended by *P*_vib_ [Eqs. (7), (8)]. (c) *T*_osc1_(*R*_max_) for free liquid (large cuvette), free liquid surface in the small cuvette, and weak confinement. (d) Peak amplitude of the wave *P*_vib_(t) originating from the wall vibrations plotted as a function of *R*_max_.

In the fitting procedure, we use a least-square fitting scheme, while varying *α* over a range from 10^6^ to 10^7^ Pa with a linear step size of 10^6^ Pa, and *t*_0_ over a range from 50 μs to 75 μs with a step size of 1 μs. For fitting the damping coefficient *τ*, we evaluate how strongly the exponential decay term exp[-(*t* - *t*_0_)/*τ*] in Eq. (7) decreases after one period of *T*_wall_. For this purpose, exp(-*T*_wall_/*τ*) is tuned in the range of 0.1 to 0.9 with a step size of 0.01. Fitting was based on 84 experimental *R*(*t*) curves for the case with weak confinement and 54 curves for the free-surface condition, all with *R*_max_ > 400 μm (*γ >* 0.04). The best fits were achieved with *α* = 5 × 10^6^ Pa, *t*_0_ = 60 μs and *τ* = 51.3 μs for weak confinement, and with *α* = 2 × 10^6^ Pa, *t*_0_ = 54 μs and *τ* = 40.7 μs for the free-surface condition. The simulation results in comparison with experimental data are presented in Fig. 10.

With free surface and weak confinement, the agreement is generally very good both for the *R*(*t*) curves during the first oscillation [Fig. 10(a) and (b)] and for the *T*_osc1_(*R*_max_) curves in Fig. 10(c). However, for weak confinement, a deviation is observed in the collapse phase of large bubbles with *R*_max_ > 500 μm. We attribute this deviation to the formation of secondary cavitation bubbles around the primary bubble by the tensile stress arising from cuvette wall vibrations. The formation of secondary bubbles reduces the tensile stress amplitude, which leads to a weaker collapse prolongation and re-expansion of the primary bubble. With free surface, *P*_vib_ is smaller than for weak confinement as shown in Fig. 10 (d), and no secondary microbubbles are produced. Therefore, the agreement between experiments and simulations is here very good.

Fig. 10(d) presents the fitted peak amplitudes of *P*_vib_ from Figs. 10(a) and (b) as a function of the maximum radius of the primary laser-induced bubble. With free surface, the amplitude *A* increases from approximately 40 kPa at *R*_max_ = 200 μm to 120 kPa at *R*_max_ = 600 μm, and with weak surface confinement, it ranges between 100 and 300 kPa. These values are consistent with the observation of secondary cavitation bubbles around large primary bubbles under weak surface confinement, and resemble the results of transducer measurements of the tensile stress reported by Orimi et al. [34]. It should be noticed that *P*_vib_ under weak confinement is larger than *P*_comp_ under strong confinement conditions in the entire range of investigated bubble sizes.

### 5.3 Bubble re-oscillations under weak confinement

**Fig. 11:**
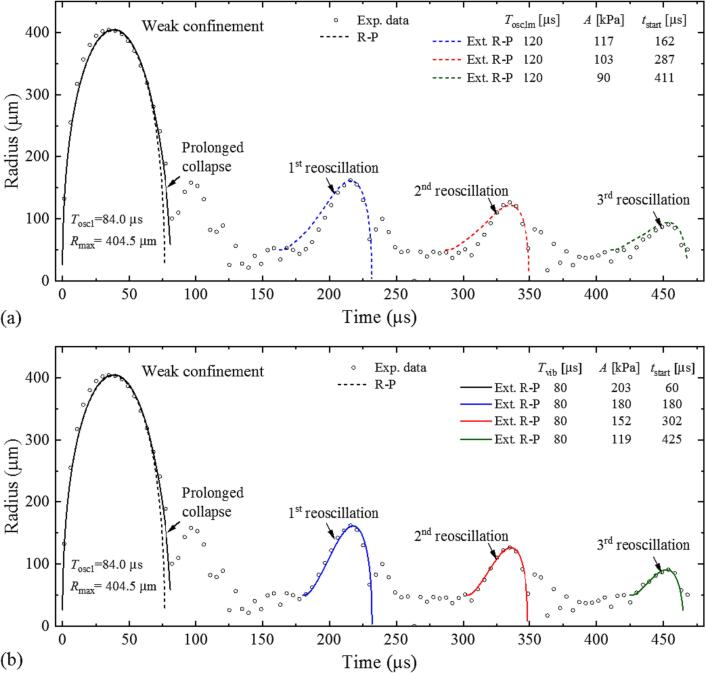
Simulations of the re-oscillation dynamics with weak confinement in comparison with experimental data. *E*_L_ = 850 μJ, *T*_osc1_ = 84.0 μs, *R*_max_ = 404.5 μm. (a) Results obtained under the assumption that the *i*-th re-oscillation is directly caused by the *i*-th tensile phase of a single pressure wave arising from the movement of the liquid mass in the cuvette corners. (b) Results obtained for independent excitation of each re-oscillation by pressure waves with the same properties as those produced by cuvette wall oscillations.

The simulations for weakly confined bubble dynamics in section 5.2 showed that the pressure wave produced by cuvette wall vibrations decays rapidly, with a time constant of 51.3 μs. Its amplitude drops to 20 % within a single 80-µs long oscillation period, making it unlikely for subsequent negative phases to cause large re-oscillations of mid-sized bubbles after *t* ≥ 200 µs as observed in Fig. 4(d) and 5(b). Moreover, the time interval between adjacent re-oscillations of the bubble is ≈120 μs, which is inconsistent with the period of the wall vibrations of 80 μs. This suggests that the re-oscillations are caused by another periodic motion having a period of ∼ 120 μs and a slower decay. In section 4.4. we have already introduced bubble-induced liquid movement in the cuvette corners as possible additional driving mechanism for an oscillating pressure wave. This assumption is now tested by numerical simulations. The results for an experimental *R*(*t*) curve with pronounced re-oscillation peaks are shown in Fig. 11.

In a first series of simulations, we assumed that the *i*-th re-oscillation of the bubble is directly caused by the *i*-th tensile phase of a single pressure wave arising alone from the movement of the liquid mass in the cuvette corners, as described by Eq. (9). The oscillation period was set to *T*_osc,lm_ = 120 μs, corresponding to the time separation of the re-oscillation peaks. We assumed that the bubble has zero kinetic energy before the start of each re-oscillation and its expansion is only driven by an extra pressure wave. Zero kinetic energy is simulated by setting *R*_n_ = *R*_0_. Since the value of *R*_0_ around *t* = 200 µs in experimental *R*(*t*) curves is about 50 µm, we set *R*_0_ = 50 μm. The peak pressure amplitude *A* and the exponential decay part exp(-*T*_osc,lm_ /*τ*) were tuned to find the best fit for all three maximum radii of the re-oscillations of the bubble using a least-squares fitting scheme. We varied the amplitude from 50 kPa to 500 kPa with a step size of 1 kPa, and the exponential decay part exp(-*T*_osc,lm_ /*τ*) from 0.5 to 0.95 with a step size of 0.01. All three re-oscillations were fitted individually because the R-P model cannot correctly track several oscillations in a sequence. The best fit for the three maximum radii of the re-oscillations was achieved with *A* = 117 kPa and exp(-*T*_osc,lm_ /*τ*) = 0.88, and the simulation results are shown in Fig. 11(a). It is obvious that the simulated re-oscillation maxima are broader than the maxima in the experimental *R*(*t*) curve. When pressure amplitude and decay constant were tuned to match the maximum radius of the bubble, the profile of the simulated radius-time curve remained almost unchanged and the maxima were always broader than in the experimental curve.

In a second series of simulations, we linked the pressure waves to the liquid mass movement in the cuvette corners. We introduced individual pressure waves with 120 µs time separation, with *R*_0_ = 50 μm and *R*_n_ = *R*_0_ at the beginning of each re-oscillation. The period of the external pressure waves was set to 80 μs and the decay constant to 51.3 μs, as in the simulations for the feedback from cuvette wall vibrations in section 5.2. The amplitude *A* of each wave was tuned to match the maximum radius of the corresponding re-oscillation of the bubble. The results are depicted in Fig. 11 (b). We find a surprisingly good match between simulations and experimental data. The predicted peak amplitudes of the individual extra-pressure waves decreases from 180 kPa in the first re-oscillation through 152 kPa in the second re-oscillation to 119 kPa in the third oscillation.

The good fit in Fig. 11(b) suggests that the feedback from liquid mass oscillations in the cuvette corners may be linked to cuvette wall vibrations. The liquid mass oscillation arises from the liquid volume reduction Δ*V* = 4π*R*^3^(*t*)/3 below the transducer surface that is induced by the expansion of the laser-induced bubble and exhibits an eigenperiod of ≈ 120 μs. It induces overall pressure fluctuations in the cuvette, which drive strongly damped wall vibrations. These vibrations cause alternating pressure waves *P*_vib_(*t*), which are focused towards the cuvette center and excite the bubble re-oscillations. As already mentioned in section 4.3, the strength of the vibrational pressure at the cuvette center is much greater than the amplitude of the overall pressure fluctuations in the liquid, which initiate the wall vibrations. This effect explains the possibility of seemingly ‘solitary’ re-oscillations. Fig. 5(b) shows that their occurrence is restricted to intermediate bubble sizes. The detailed conditions under which the interplay between liquid-mass oscillations and wall vibrations leads to large re-oscillations are likely linked to the relative phases between the individual sources of pressure fluctuations and remain to be investigated in future.

## 6 Conclusions and outlook

We investigated the cavitation dynamics in a small container with elastic thin walls and free or partially confined surface experimentally and by numerical investigations. Bubbles with 200 µm to 600 µm maximum radius were produced by focusing 6-ns laser pulses into the center of a cuvette with 10 × 10 mm^2^ cross section. The liquid surface was either free or confined by a piston-shaped transducer with 9.25 mm diameter. Different degrees of confinement were realized by filling the liquid up to the transducer surface or 18 mm higher up to the top of the cuvette. For reference, some experiments were performed in a larger cuvette with acoustically absorbing walls that simulated “infinitely” extended liquid. The bubble dynamics was simultaneously recorded by high-speed photography, detection of the pressure transients emitted upon bubble generation and collapse, and cw probe beam measurements of light scattering by the bubble. Simultaneous single-shot recording of radius-time curves and oscillation times enabled to perform detailed investigations of the bubble dynamics as a function of bubble size and type of acoustic feedback from the elastic bubble walls and the degree of surface confinement.

In future experiments, it would be of interest to perform time-resolved measurements of the cuvette wall vibrations, for example by means of a laser Doppler vibrometer or probe beam deflection on a quadrant diode. Such measurements can provide *direct* information about the vibration period of the wall and potential higher oscillation modes, whereas we merely deduced *T*_wall_ from the *T*_osc2_/*T*_osc1_ curve. Moreover, time-resolved measurements enable to evaluate the contributions of breakdown shock wave impact and bubble expansion on the excitation of wall vibrations because the shock wave impact is expected to produce an impulsive acceleration at an early time, while the acceleration by the bubble expansion will be more continuous. Finally, vibration measurements may help to further elucidate the origin of the late re-oscillations for mid-sized bubbles under weak confinement.

The bubble dynamics was numerically simulated using a Rayleigh-Plesset model of laser induced bubble oscillations extended by terms describing the acoustically mediated feedback from liquid confinement, elastic wall vibrations, and liquid movement in the cuvette corners.

The bubble oscillations were found to be approximately spherical as long as no secondary cavitation occurred, where tensile stress produced transient bubble clusters. Bubble expansion was always similar to the dynamics in free liquid, and mainly the collapse phase and subsequent oscillations were influenced by feedback from the bubble’s surrounding. For sufficiently large bubbles, strong confinement led to a slight reduction of maximum bubble size and to a pronounced reduction of the oscillation time. Both effects increased with bubble size. The pressure increase resulting from the bubble expansion and pressure waves from liquid mass oscillations in the cuvette corners both induce cuvette wall vibrations. These vibrations produce pressure waves with alternating negative and positive fluctuations around the ambient pressure value. The waves are focused into the cuvette center, where the laser-induced bubble is located. The interaction of the pressure waves with the bubble results in a prolongation of its collapse phase and, depending on circumstances, an enlargement of the second oscillation or time-delayed re-oscillations. The details of the bubble dynamics during first collapse and later oscillations depend in a complex manner on the degree of surface confinement and on bubble size.

Numerical simulations of the first bubble oscillation provided a very good agreement with experimental *R*(*t*) and *T*_osc_ data. They suggest that cuvette wall vibrations induced by confinement-related pressure rise cause a prolongation of the first oscillation, while the vibrations causing re-oscillations are linked to liquid mass movement in the cuvette corners. The Rayleigh-Plesset model used in the present study cannot track the bubble dynamics beyond the first collapse because it does not consider liquid compressibility and the damping of bubble oscillations by shock wave emission. Future modeling considering liquid compressibility may enable to portray the interplay between pressure evolution inside the bubble and external acoustic feedback over a longer time.

The pressure variations arising from the breakdown-induced wall vibrations were determined indirectly by matching numerical simulations of the bubble dynamics to the experimental results. The pressure values obtained this way agree well with the results of needle hydrophone measurements reported in Ref. [34]. However, in future experiments, it would be desirable to perform direct measurements of the pressure in the bubble’s vicinity by means of a sensitive small-area detector (e.g. a needle hydrophone) directed towards the site of the laser-induced bubble. For geometrical reasons, this was not possible in the present setup. It could be done by utilizing a normal piston instead of a large area transducer and inserting the needle hydrophone in the middle of the piston such that it points at the location of bubble generation.

Altogether, our study revealed a surprisingly strong and rich interaction between the bubble and its surroundings that may open a new field of investigations. Our results help to identify the prerequisites for avoiding unwanted feedback on laser-induced bubble oscillations from the environment if spherical dynamics shall be investigated. With weak and strong surface confinement, we observed a significant influence on *T*_osc2_/*T*_osc1_, when *T*_osc1_ was larger than the half of the wall vibration period, *T*_wall_ (Fig. 7). Thus, the investigated bubbles must be at least small enough relative to the container size to fulfil the condition *T*_osc1_ < *T*_wall_/2. Moreover, our findings on the variety of possible feedback effects and mechanisms may be relevant for a variety of applications such as laser angioplasty [35], [36], laser lithotripsy [37], [38], [39], and laser-induced cavitation in microfluidics, which has been employed for cell sorting [59], [60] and cell membrane poration [41], [61] as well as for rapid mixing [40] and micro-pumping [62]. In many of these scenarios, laser-induced cavitation bubbles are generated in a semi-sealed domain such as tubes and narrow channels, where the size of the generated bubble is not much smaller than the size of the container. In such environments, the interactions between bubble oscillations and the rigid or viscoelastic wall response can influence the bubble dynamics in a complex way. This influence can contribute to the desired effects but also evoke undesired side effects, and a better understanding of possible effects is useful for the optimization of the cavitation-mediated applications. In this context, it will be of interest to perform experiments with different mechanical properties of the wall material that mimic biological tissues or resemble those used in microfluidics.

## Funding

National Natural Science Foundation of China (62005210, 62175198, 62005325); Open Research Fund of State Key Laboratory of Transient Optics and Photonics (SKLST202004); Sino-German Mobility Programme (M-0063); Sino-German (CSC-DAAD) Postdoc Scholarship Program (2022; 57607866).

## CRediT authorship contribution statement

**Lei Fu:** Conceptualization, Methodology, Software, Investigation, Writing – original draft, Visualization. **Xiao-Xuan Liang:** Conceptualization, Methodology, Formal analysis, Writing – original draft, Visualization. **Sijia Wang:** Resources. **Siqi Wang:** Investigation, Software. **Ping Wang:** Investigation. **Zhenxi Zhang:** Project administration. **Jing Wang:** Investigation. **Alfred Vogel:** Conceptualization, Writing – review & editing, Supervision. **Cuiping Yao:** Supervision, Funding acquisition, Project administration.

## Declaration of Competing Interest

The authors declare that they have no known competing financial interests or personal relationships that could have appeared to influence the work reported in this paper.

## References

[1] Lauterborn W., Kurz T. (2010). Physics of bubble oscillations. Rep. Prog. Phys..

[2] Liang X.-X., Linz N., Freidank S., Paltauf G., Vogel A. (2022). Comprehensive analysis of spherical bubble oscillations and shock wave emission in laser-induced cavitation. J. Fluid Mech..

[3] Denner F., Schenke S. (2023). Modeling acoustic emissions and shock formation of cavitation bubbles. Phys. Fluids.

[4] Wen H., Yao Z., Zhong Q., Tian Y., Sun Y., Wang F. (2023). Energy partitioning in laser-induced millimeter-sized spherical cavitation up to the fourth oscillation. Ultrason. Sonochem..

[5] Brenner M.P., Hilgenfeldt S., Lohse D. (2002). Single-bubble sonoluminescence. Rev. Mod. Phys..

[6] Vogel A. (1997). Nonlinear absorption: Intraocular microsurgery and laser lithotripsy. Phys. Med. Biol..

[7] Vogel A., Venugopalan V. (2003). Mechanisms of pulsed laser ablation of biological tissues. Chem. Rev..

[8] Vogel A., Noack J., Huttman G., Paltauf G. (2005). Mechanisms of femtosecond laser nanosurgery of cells and tissues. Appl. Phys. B-Lasers Opt..

[9] Hutson M.S., Ma X.Y. (2007). Plasma and cavitation dynamics during pulsed laser microsurgery in vivo. Phys. Rev. Lett..

[10] Chung S.H., Mazur E. (2009). Surgical applications of femtosecond lasers. J. Biophotonics.

[11] Blake J.R., Gibson D.C. (1987). Cavitation bubbles near boundaries. Annu. Rev. Fluid Mech..

[12] Vogel A., Lauterborn W., Timm R. (1989). Optical and acoustic investigations of the dynamics of laser-produced cavitation bubbles near a solid boundary. J. Fluid Mech..

[13] Brujan E.A., Nahen K., Schmidt P., Vogel A. (2000). Dynamics of laser-induced cavitation bubbles near an elastic boundary used as a tissue phantom. Nonlinear Acoust. Turn Millennium.

[14] Brujan E.A., Nahen K., Schmidt P., Vogel A. (2001). Dynamics of laser-induced cavitation bubbles near elastic boundaries: influence of the elastic modulus. J. Fluid Mech..

[15] Bempedelis N., Zhou J., Andersson M., Ventikos Y. (2021). Numerical and experimental investigation into the dynamics of a bubble-free-surface system. Phys. Rev. Fluids.

[16] Saade Y., Jalaal M., Prosperetti A., Lohse D. (2021). Crown formation from a cavitating bubble close to a free surface. J. Fluid Mech..

[17] Lindau O., Lauterborn W. (2003). Cinematographic observation of the collapse and rebound of a laser-produced cavitation bubble near a wall. J. Fluid Mech..

[18] Lechner C., Lauterborn W., Koch M., Mettin R. (2020). Jet formation from bubbles near a solid boundary in a compressible liquid: Numerical study of distance dependence. Phys. Rev. Fluids.

[19] Gonzalez-Avila S.R., Denner F., Ohl C.D. (2021). The acoustic pressure generated by the cavitation bubble expansion and collapse near a rigid wall. Phys. Fluids.

[20] Bußmann A., Riahi F., Gökce B., Adami S., Barcikowski S., Adams N.A. (2023). Investigation of cavitation bubble dynamics near a solid wall by high-resolution numerical simulation. Phys. Fluids.

[21] Bokman G.T., Biasiori-Poulanges L., Meyer D.W., Supponen O. (2023). Scaling laws for bubble collapse driven by an impulsive shock wave. J. Fluid Mech..

[22] Gonzalez-Avila S.R., Klaseboer E., Khoo B.C., Ohl C.D. (2011). Cavitation bubble dynamics in a liquid gap of variable height. J. Fluid Mech..

[23] Brujan E.-A., Noda T., Ishigami A., Ogasawara T., Takahira H. (2018). Dynamics of laser-induced cavitation bubbles near two perpendicular rigid walls. J. Fluid Mech..

[24] Han B., Zhu R.H., Guo Z.Y., Liu L., Ni X.W. (2018). Control of the liquid jet formation through the symmetric and asymmetric collapse of a single bubble generated between two parallel solid plates. Eur. J. Mech. B-Fluids.

[25] Tagawa Y., Peters I.R. (2018). Bubble collapse and jet formation in corner geometries. Phys. Rev. Fluids.

[26] Li S.M., Zhang A.M., Wang Q.X., Zhang S. (2019). The jet characteristics of bubbles near mixed boundaries. Phys. Fluids.

[27] Brujan E.-A., Zhang A.M., Liu Y.-L., Ogasawara T., Takahira H. (2022). Jetting and migration of a laser-induced cavitation bubble in a rectangular channel. J. Fluid Mech..

[28] Martynov S., Stride E., Saffari N. (2009). The natural frequencies of microbubble oscillation in elastic vessels. J. Acoust. Soc. Am..

[29] Wang S.P., Wang Q.X., Leppinen D.M., Zhang A.M., Liu Y.L. (2018). Acoustic bubble dynamics in a microvessel surrounded by elastic material. Phys. Fluids.

[30] Luo X., Chen T., Xiao W., Yao X., Liu J. (2022). The dynamics of a bubble in the internal fluid flow of a pipeline. Phys. Fluids.

[31] Vincent O., Marmottant P., Gonzalez-Avila S.R., Ando K., Ohl C.D. (2014). The fast dynamics of cavitation bubbles within water confined in elastic solids. Soft Matter.

[32] Vincent O., Marmottant P. (2017). On the statics and dynamics of fully confined bubbles. J. Fluid Mech..

[33] Wang Q.X. (2017). Oscillation of a bubble in a liquid confined in an elastic solid. Phys. Fluids.

[34] Orimi H.E., Arreaza L., Narayanswamy S., Boutopoulos C. (2021). Self-limited nanosecond laser-induced bubble growth in sealed containers. Appl. Phys. Lett..

[35] Vogel A., Engehardt R., Behnle U., Parlitz U. (1996). Minimization of cavitation effects in pulsed laser ablation illustrated on laser angioplasty. Appl. Phys. B-Lasers Opt..

[36] Van Leeuwen T.G., Meertens J.H., Velema E., Post M.J., Borst C. (1993). Intraluminal vapor bubble induced by excimer laser-pulse causes microsecond arterial dilation and invagination leading to extensive wall damage in the rabbit. Circulation.

[37] Brinkmann R., Hansen C., Mohrenstecher D., Scheu M., Birngruber R. (1996). Analysis of cavitation dynamics during pulsed laser tissue ablation by optical on-line monitoring. IEEE J. Sel. Top. Quantum Electron..

[38] Mohammadzadeh M., Mercado J.M., Ohl C.-D. (2015). Bubble dynamics in laser lithotripsy. J. Phys. Conf. Ser..

[39] Fried N.M. (2018). Recent advances in infrared laser lithotripsy. Biomed. Opt. Express.

[40] Hellman A.N., Rau K.R., Yoon H.H., Bae S., Palmer J.F., Phillips K.S., Allbritton N.L., Venugopalan V. (2007). Laser-induced mixing in microfluidic channels. Anal. Chem..

[41] Sankin G.N., Yuan F., Zhong P. (2010). Pulsating tandem microbubble for localized and directional single-cell membrane poration. Phys. Rev. Lett..

[42] Anna S.L. (2016). Droplets and bubbles in microfluidic devices. Annu. Rev. Fluid Mech..

[43] Vogel A., Linz N., Freidank S., Paltauf G. (2008). Femtosecond-laser-induced nanocavitation in water: Implications for optical breakdown threshold and cell surgery. Phys. Rev. Lett..

[44] Smith W.L., Bechtel J.H., Bloembergen N. (1975). Dielectric-Breakdown Threshold and Nonlinear-Refractive-Index Measurements with Picosecond Laser Pulses. Phys Rev B.

[45] Vogel A., Nahen K., Theisen D., Birngruber R., Thomas R.J., Rockwell B.A. (1999). Influence of optical aberrations on laser-induced plasma formation in water and their consequences for intraocular photodisruption. Appl. Opt..

[46] Fu L., Wang S., Xin J., Wang S., Yao C., Zhang Z., Wang J. (2018). Experimental investigation on multiple breakdown in water induced by focused nanosecond laser. Opt. Express.

[47] Brujan E.A., Vogel A. (2006). Stress wave emission and cavitation bubble dynamics by nanosecond optical breakdown in a tissue phantom. J. Fluid Mech..

[48] Lohmann S., Olmes A., Lubatschowski H., Ertmer W. (1996). Characterization of laser-induced pressure transients by means of piezoelectric PVDF films. Laser-Tissue Interaction Tissue Opt..

[49] Olmes A., Lohmann S., Lubatschowski H., Ertmer W. (1997). An improved method of measuring laser induced pressure transients. Appl. Phys. B-Lasers Opt..

[50] Vogel A., Lauterborn W. (1988). Acoustic transient generation by laser-produced cavitation bubbles near solid boundaries. J. Acoust. Soc. Am..

[51] Alster M. (1972). Improved Calculation F Resonant Frequencies of Helmholtz Resonators. J Sound Vib.

[52] Wikipedia, Helmholtz resonance. https://en.wikipedia.org/wiki/Helmholtz_resonance.

[53] Thompson D., Nguyen A., Morony M. Resonating Wine Glasses. https://www.researchgate.net/publication/325590328_Resonating_Wine_Glasses.

[54] Plesset M.S. (1949). The dynamics of cavitation bubbles. J. Appl. Mech.-Trans. ASME.

[55] Plesset M.S., Prosperetti A. (1977). Bubble dynamics and cavitation. Annu. Rev. Fluid Mech..

[56] N. Linz, S. Freidank, X.-X. Liang, J. Noack, G. Paltauf, A. Vogel, Roles of tunneling, multiphoton ionization, and cascade ionization for optical breakdown in aqueous media, Final Report AOSR Grant FA 8655-05-1-3010 (2009), https://apps.dtic.mil/sti/citations/ADA521817.

[57] Vogel A., Noack J., Nahen K., Theisen D., Busch S., Parlitz U., Hammer D.X., Noojin G.D., Rockwell B.A., Birngruber R. (1999). Energy balance of optical breakdown in water at nanosecond to femtosecond time scales. Appl. Phys. B-Lasers Opt..

[58] Vogel A., Busch S., Parlitz U. (1996). Shock wave emission and cavitation bubble generation by picosecond and nanosecond optical breakdown in water. J Acoust Soc Am.

[59] Chen Y., Wu T.H., Kung Y.C., Teitell M.A., Chiou P.Y. (2013). 3D pulsed laser-triggered high-speed microfluidic fluorescence-activated cell sorter. Analyst.

[60] Chen Y., Chung A.J., Wu T.H., Teitell M.A., Di Carlo D., Chiou P.Y. (2014). Pulsed laser activated cell sorting with three dimensional sheathless inertial focusing. Small.

[61] Li Z.G., Liu A.Q., Klaseboer E., Zhang J.B., Ohl C.D. (2013). Single cell membrane poration by bubble-induced microjets in a microfluidic chip. Lab Chip.

[62] Cao K.J., Liu Y., Qu S.L. (2017). Quantitative microfluidic delivery based on an optical breakdown-driven micro-pump for the fabrication of fiber functional devices. Opt. Express.

